# Roles of Cadherin2 in Thyroid Cancer

**DOI:** 10.3389/fonc.2022.804287

**Published:** 2022-06-09

**Authors:** Yun Chen, Chaojin Hong, Qihao Zhou, Zhiquan Qin

**Affiliations:** Cancer Center, Department of Medical Oncology, Zhejiang Provincial People’s Hospital, Affiliated People’s Hospital, Hangzhou Medical College, Hangzhou, China

**Keywords:** CDH2, thyroid cancer, FRMD3, immune cell infiltration, immune checkpoint

## Abstract

**Background:**

The majority of drug-resistant cells in Thyroid cancer (THCA) tend to exhibit an Epithelial mesenchymal transition (EMT) phenotype, and abnormal expression of the cell adhesion molecule Cadherin2 (CDH2) is a hallmark of EMT. However, the roles of CDH2 in THCA and its underlying mechanisms are unknown.

**Methods:**

We analyzed the CDH2 expression in The Cancer Genome Atlas (TCGA) database and screened for genes positively associated with CDH2. Small interfering RNA and cell transfection were used for knocking down CDH2 in THCA cells, cell counting kit-8 (CCK-8) assay and immunofluorescence to detect cell proliferation. Binding miRNAs of CDH2 and CDH2-associated genes were predicted using the Encyclopedia of RNA Interactomes (ENCORI) database. The expression of genes in clinical THCA tissues was investigated from the Human Protein Atlas (HPA) database and validated by qRT-PCR. We conducted the cell functions pathways of CDH2 and CDH2-associated gene FRMD3 by Gene Ontology (GO) and Kyoto Encyclopedia of Genes and Genomes (KEGG) analysis. We also showed the correlation between CDH2 and FRMD3 expression and tumor immune infiltration.

**Results:**

The expression of CDH2 was significantly higher in THCA tumor tissues compared to normal tissues. Moreover, there were strongly associations of CDH2 expression with the stages T and N. Cellular function assays showed that CDH2 exerted its growth-promoting activity of THCA. To better understand how CDH2 was regulated in THCA, we sought genes associated with CDH2. Correlation analysis revealed that there were negative correlations between genes (CDH2, FRMD3) and miRNAs (hsa-miR-410-3p, hsa-miR-411-5p, hsa-miR-299-5p). Moreover, CDH2 and FRMD3 expression were significantly higher in tumor tissues than in normal tissues, while hsa-miR-410-3p, hsa-miR-411-5p and hsa-miR-299-5p were significantly decreased in tumor tissues compared with normal tissues in THCA. GO and KEEG results showed that CDH2 and FRMD3 were strongly associated with immune-related functions. High expression of CDH2 and FRMD3 was linked to the suppression of immune cells. There were strong negativity correlations between CDH2, FRMD3 and T-cell exhaustion factors.

**Conclusion:**

Our data indicated that CDH2 and CDH2-related gene FRMD3 might have the critical effects on altering tumors becoming ‘cold tumors’ eventually leading to immune checkpoint inhibitor resistance.

## Introduction

Thyroid cancer (THCA) is a common malignant tumor in the endocrine system (1). The etiology is related to radiation, dietary factors (high iodine or iodine deficiency diet), increased estrogen production, genetics, or other benign thyroid diseases such as nodular goiter or thyroid adenoma (2). Differentiated thyroid carcinoma (DTC) is the most common type of THCA, and it can be classified into papillary thyroid carcinoma (PTC), follicular thyroid carcinoma (FTC) and and medullary thyroid carcinoma (MTC) and undifferentiated THCA (3). PTC is the most common among THCA, accounting for about 85%, and is considered to be one of the malignancies with the best clinical prognosis due to its weak invasive metastatic ability (4). FTC is the second most common, which accounts for about 10-15% of THCA, but it is much more aggressive than DTC and has a poorer prognosis than PTC due to its frequent hematologic metastases (5).

Currently, complete thyroidectomy is mostly used for initial treatment, and radioiodine therapy is administered when recurrence and distant metastases have occurred (6). A proportion of patients (5-10%) develop metastases outside the thyroid or cervical lymph nodes, despite the overall good clinical prognosis of THCA, and these patients have a poorer prognosis. The transformation of THCA from an inert tumor to an aggressive malignancy is characterized by a complex series of genetic alterations, including BRAFV600E mutations (7) and TERT rearrangements (8). Aberrant alterations in these genes ultimately lead to aberrant activation of tumor-associated signaling pathways such as MAPK and PI3K/Akt/mTOR (9, 10). Despite the increasing number of studies in recent years that have identified biomarkers enabling the diagnosis of THCA, however, approximately 10% of THCA still have reduced survival rates due to local recurrence and distant metastases. Therefore, it is essential to identify reliable prognostic markers for patients with THCA to enhance the surveillance, prompt treatment and prolong the survival time.

Epithelial mesenchymal transition (EMT) is regarded as the preliminary and essential process followed by epithelial cells losing their cell polarity and intercellular adhesion and acquiring migratory and invasive properties (11). The EMT process is modulated by a variety of calcium-dependent molecules involved in epithelial maintenance, such as calmodulin (e.g., E-calmodulin and N-cadherin) (12). Cadherin is known to be involved in the maintenance of tissue epithelial structure and represents an important contribution to embryonic development and the maintenance of normal tissue architecture (13). N-cadherin (also known as Cadherin2 (CDH2)) is an adhesion molecule that is frequently overexpressed in several types of cancers, including breast and colorectal cancers, and is considered to be an inducer of EMT, which serves an important function in the early invasion and metastasis of cancers (14, 15). Recent studies have revealed that CDH2 facilitates the development and progression of THCA by regulating major signaling pathways and EMT processes and may be a potential therapeutic target for THCA (16, 17). However, little literature has reported how CDH2 specifically affects the progression of THCA.

MicroRNAs (miRNAs) are a class of non-coding RNA molecules of approximately 22 nucleotides in length that regulate gene expression by degrading mRNA (18, 19). It has been demonstrated that miRNAs exert their biological functions by participating in the regulation of their downstream gene translation processes (20). It was found that a single mRNA can recognize multiple mRNA targets, and one mRNA target could be recognized by multiple miRNAs (21). miRNA targeting of complementary mRNAs leads to degradation of the target mRNA, and miRNAs can induce the RNA-induced silencing complex (RISC) to down-regulate gene expression at the post-transcriptional level, culminating in mRNA degradation or translational repression (20). Exploring the ceRNA network of CDH2 is facilitated to understand the regulatory mechanism of CDH2.

Recently, there is evidence that the immune microenvironment associated with THCA may serve crucial functions in tumor progression, with potentially immunotherapeutic implications for the advanced and metastatic THCA (22). Immune checkpoint (ICP) therapeutic monoclonal antibodies exhibit essential therapeutic benefits in a variety of tumors, including THCA (23, 24).. In the present study, we investigated the relevance of CDH2 on the immune microenvironment and ICP of THCA.

## Materials and Methods

### Data Acquisition From the Cancer Genome Atlas (TCGA) Database

The RNAseq or miRNAseq data from the pan-cancer, including THCA sample, was downloaded from TCGA database (https://portal.gdc.cancer.gov/). Then the data in Fregments Per Kilobase per Million (FPKM) format was converted to transcripts per million reads (TPM) format and the log2 transformation was carried out using R language. The corresponding clinical information, such as stage and clinical outcomes of THCA samples was obtained from the UCSC Xena browser (https://xena.ucsc.edu/).

### Bioinformatics Analysis

To investigate the expression of genes or miRNAs in THCA patients, 510 tumor tissues and 58 adjacent normal tissues were used analysis with the expression of genes, and 514 tumor tissues and 59 adjacent normal tissues were utilized and analysed for the levels of miRNAs. The R package ggplot2 was applied for visualization and the Wilcoxon rank sum test was used to detect the expression of target genes in tumor and normal tissues. For the analysis of paired samples, only the paired samples were reserved, the ggplot2 package was used for visualization, and the paired samples t-test was performed for statistical analysis.

In ROC curve analysis, the R package pROC was used for analysis and the ggplot2 package was provided for results visualization. The horizontal coordinates of the results are the False Positive Rate (FPR) and the vertical coordinates are the True Positive Rate (TPR). The area under the ROC curve is between 0.5 and 1. The closer the AUC is to 1, the better the diagnosis. The AUC is of lower accuracy at 0.5 to 0.7, moderate accuracy at 0.7 to 0.9, and higher accuracy at AUC above 0.9.

To detect CDH2-related genes, the R package stat was used for results visualization and pearson was utilized for statistical analysis. To investigate the association between the key genes and miRNAs, the R package ggplot2 package was applied for visualization and pearson was provided for statistical analysis.

We further investigate the relationship between the Hub gene expression and THCA prognosis The R package survminer was used for the visualization and the R package survival was applied for the statistical analysis of the survival data. Cox regression was provided for statistical analysis and P < 0.05 was considered statistically significant.

For Gene Ontology (GO) and Kyoto Encyclopedia of Genes and Genomes (KEGG) analysis, THCA patients were divided into high and low expression groups according to the median values of the expression of CDH2 or FRMD3. Fold change (FC) and false discovery rate (FDR) were used to screen for differentially expressed genes (DEGs). The R package DESeq2 was used to extract the differential genes and |log(FC)| ≥1 and FDR < 0.05 were defined as the screening criteria for DEGs (25). The R package ClusterProfiler was performed for GO and KEGG analysis. P values were adjusted using the BH method.

The correlation between the expression levels of CDH2 or FRMD3 and immune cell infiltration was performed using ssGSEA analysis by the GSVA R package (26). The Immune cells included aDC (activated DC); B cells; CD8 T cells; Cytotoxic cells; DC; Eosinophils; iDC (immature DC); Macrophages; Mast cells; Neutrophils; NK CD56bright cells; NK CD56dim cells; NK cells; pDC (Plasmacytoid DC); T cells; T helper cells; Tcm (T central memory); Tem (T effector memory); Tfh (T follicular helper); Tgd (T gamma delta); Th1 cells; Th17 cells; Th2 cells; Treg (27). The Spearman analysis was used as a statistical difference analysis.

### Prediction of MiRNAs Binding to the Genes

The ENCORI database (https://starbase.sysu.edu.cn/) was used to investigate the binding miRNAs of the key genes. The data in the ENCORI database are derived from CLIP-seq and demonstrate miRNA-target interactions by predicting the intersection of miRNA targets with the binding sites of Ago proteins, and this database provides results from seven prediction programs (PITA, RNA22, miRmap, DIANA-microT, miRanda, PicTar and TargetScan) (28). The miRNA-mRNA was predicted using the miRNA targeting base factor approach and the miRNA-mRNA ceRNA network was established. The establishment of miRNA-mRNA ceRNA network requires two conditions, one is the negative correlation of the predicted miRNA with the target gene; and the other is the low expression of the predicted miRNA in the tumor tissues.

### The Validation of Protein Expression of CDH2 and FRMD3

The protein levels of CDH2 and FRMD3 in normal and THCA tumor tissues were verified using the Human Protein Atlas database (HPA, https://www.proteinatlas.org/). The HPA database is a protein expression profiling-based database that detects protein expression levels of genes in patient specimens by immunohistochemistry (IHC) (29–31). The HPA database provides the information on the protein expression levels of genes in normal and tumor tissues and the cellular localization of proteins.

### Cell Culture and Cell Transfection

The THCA cells were cultured in Dulbecco’s Modified Eagle Media (DMEM) (Gibco) containing 10% Fetal Bovine Serum (FBS) (Gibco) and 1% penicillin and streptomycin (Gibco). All cells were maintained at 37°C and 5% CO_2_. The Lipofectamine 2000 reagent was used to cell transfection according to the company’s instructions. The sequences of CDH2 siRNA (Sigma) are as follows: Sense: CUGAGUUUCUGCACCAGGUTT; Antisense: ACCUGGUGCAGAAACUCAGTT.

### Cell Proliferation Assay

Cell Counting Kit-8 (CCK-8, Beyotime Biotechnology, China) was used to determine the proliferation ability of the cells. After cell transfection, 10 μl of CCK-8 solution was added to each well of a 96-well plate and incubated at 37°C for 1 hour. After incubation, the absorbance levels were measured at 450 nm (OD450). Measurements were carried out at 0h, 24h, 48h and 72h, respectively.

### Western Blot Assay

Cells were lysed using RIPA buffer (Beyotime Biotechnology, China) and proteins were quantified using a BCA protein assay kit (ThermoFisher Scientific). A total of 30 μg of protein was separated by SDS PAGE in a 10% gel and transferred to a PVDF membrane (Millipore). After incubation with 5% fat-free milk, the membranes were incubated with primary antibody (anti-CDH2, Santa Cruz Biotechnology, sc-393933, 1:500 dilution; anti-GAPDH, abcam, ab8245, 1:1000 dilution) overnight at 4°C. The next day the membrane is incubated with secondary antibodies (Cell Signaling Technology) for 2 hours at room temperature.

### Immunofluorescence Assay

After transfection, cells were washed with PBS and fixed with 4% paraformaldehyde (PFA). Cells were permeabilized with 0.3% Triton X-100 and incubated in 5% BSA for 1 hour. Cells were then incubated with primary antibody (anti-Ki-67, abcam, ab16667, 1:500 dilution) overnight at 4°C and further incubated with secondary antibody (Invitrogen, 1:200 dilution). Cell nuclei were stained with DAPI (1:1000).

### Patients and Human THCA Tissues

The 28 THCA clinical samples were collected from Zhejiang Provincial People’s Hospital. The fresh frozen tumor and adjacent normal tissues of THCA patients were collected and preserved in RNA tissue preservation solution (Beyotime Biotechnology, China) and stored in liquid nitrogen. All samples used in this project were de-identified and all procedures were approved by the committee of Zhejiang Provincial People’s Hospital (Ethical number: 2021QT361). The pathological features of 28 THCA were shown in **Supplement Table 1**.

### RNA Isolation and Real-Time Quantitative PCR (qRT-PCR)

Total RNA from clinical tissues or cells was extracted by the Total RNA Purification Kit (Norgen Biotek) and the concentration and quality were checked by Nanodrop Lite spectrophotometer. For the levels of CDH2 and FRMD3 analysis, total 250 ng RNA was reverse transcribed to cDNA (BIO-RAD, #1708841) and then qPCR assays were performed. The fold changes of CDH2 and FRMD3 were calculated using 2^- ΔΔCt^ method. The primer sequences were as follows: CDH2 F: 5’- GGACAGTTCCTGAGGGATCA -3’, R: 5’- GGATTGCCTTCCATGTCTGT -3’ (32); FRMD3 F: 5’-TCCCCCAGCGAGCAAGAAG-3’, R: 5’-ACCCGAATATGGCCAGTCAGAATG-3’ (33); GAPDH F: 5’- ATGTTCGTCATGGGTGTGAA-3’, R: 5’- TGTGGTCATGAGTCCTTCCA-3’. The expression of CDH2 and FRMD3 was normalized by GAPDH. For the levels of has-miR-410-3p, has-miR-411-5p and has-miR-299-5p, total 250 ng RNA was reverse transcribed using miRCURY LNA RT kit (QIAGEN, 339340). Then qPCR assays were performed using miRCURY LNA SYBR Green PCR kit (QIAGEN, 339347). The miRNA primers were as follows: has-miR-410-3p, cat no MIMAT0002171; has-miR-411-5p, cat no MIMAT0003329; has-miR-299-5p, cat no MIMAT0002890 (QIAGEN). The expression of miRNAs was normalized by miR-125a. The mRNA levels of key genes and miRNAs in normal and tumor tissues were verified by qRT-PCR were analyzed by two-tailed t test.

### Statistical Analysis

R language 4.0.5 or SPSS 16.0 software were used for statistical anlaysis. Details of bioinformatics analysis can be found in methods parts. The difference between the two groups was estimated by Student’s t-test (two-tailed). P<0.05 was considered to be statistically significant.

## Results

### CDH2 Is Overexpressed in THCA

**Figure 1:**
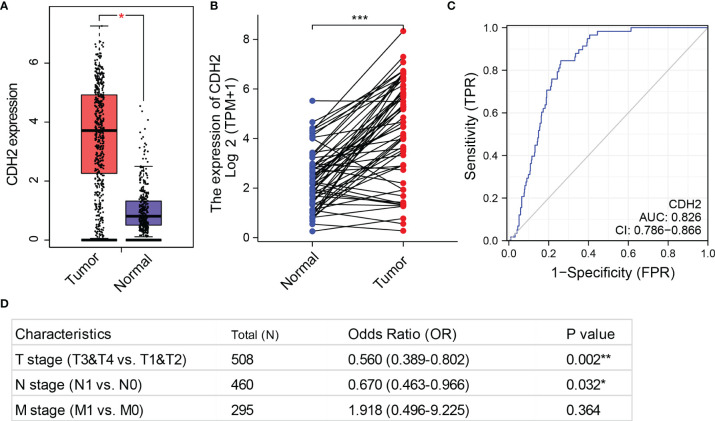
CDH2 is overexpressed in THCA. **(A)** The mRNA levels of CDH2 in TCGA tumor tissues (n=512) and matched normal tissues (normal tissues in TCGA + GTEx normal tissues, n=337) were generated by GEPIA database. **(B)** CDH2 expression in 58 paired tissues (normal and tumor tissues) in THCA. **(C)** ROC curves to predict tumor and normal outcomes in THCA. **(D)** The associations of CDH2 mRNA expression with the stages T, N and M using univariate COX regression analysis of the TCGA dataset. *P < 0.05; **P < 0.01; ***P < 0.001.

To examine the impacts of CDH2 on THCA, we first investigated the mRNA expression levels of CDH2 from THCA samples in the TCGA database. We used the GEPIA database (http://gepia.cancer-pku.cn/) to investigate the expression levels of CDH2 in THCA tumor tissues (n=512) and matched normal tissues (normal tissues in TCGA + GTEx normal tissues, n=337), and the results showed that CDH2 was significantly higher in tumor tissues than in normal tissues in THCA (**Figure 1A**). Similarly, CDH2 expression in 58 paired tissues exhibited markedly higher in tumor tissues than in normal tissues in THCA (**Figure 1B**). We also used ROC curves to predict tumor and normal outcomes, and the results showed that CDH2 had a certain accuracy in its predictive power (AUC = 0.826, CI = 0.786-0.866) (**Figure 1C**).

Next, we analyzed the associations of CDH2 mRNA expression with the stages T, N and M using univariate COX regression analysis of the TCGA dataset. CDH2 expression in stages T3 & T4 was statistically significant compared to stages T1 & T2 (p=0.002<0.01). There was a difference in the levels of CDH2 in stage N1 compared to stage N0 (P=0.032<0.05). However, CDH2 expression was not statistically different in the M stages (M0 and M1) (P=0.364>0.05) (**Figure 1D**).

### CDH2 Promotes the Growth of THCA Cells

**Figure 2:**
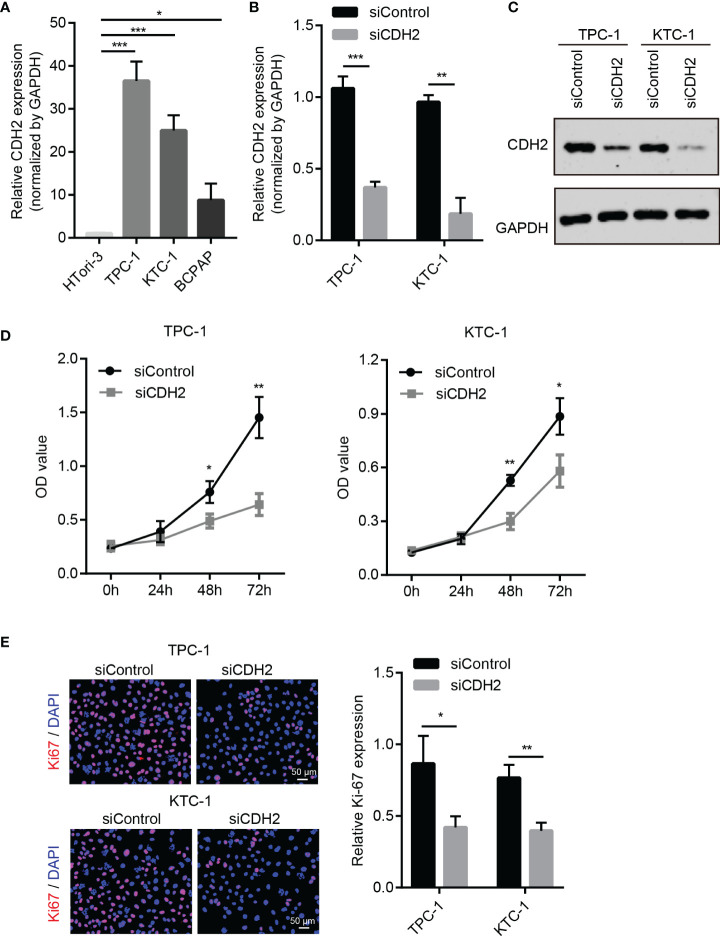
CDH2 promotes the growth of THCA cells. **(A)** The mRNA expression of CDH2 in human normal thyroid cells HTori-3 and THCA cell lines (TPC-1, KTC-1 and BCPAP) by qPCR assay. **(B)** After transfection with siRNA, the expression levels of CDH2 were examined using qPCR assay. **(C)** The protein levels of CDH2 after transfection with siRNA were detected by western blot assay. **(D)** After knocking down CDH2 expression, the proliferation ability of THCA cells TPC-1 and KTC-1 was significantly decreased using CCK8 assay. **(E)** After knocking down CDH2 expression, the Ki67 expression was tested by Immunofluorescence assay. *P < 0.05; **P < 0.01; ***P < 0.001.

We further explored the effects of CDH2 on the cell function of THCA. First, we examined the expression levels of CDH2 in human normal thyroid cells HTori-3 and THCA cell lines (TPC-1, KTC-1 and BCPAP), and the results showed that in contrast to HTori-3, CDH2 was significantly increased in different THCA cells (**Figure 2A**). To test the influence of CDH2 on the proliferation of THCA cells, we knocked down CDH2 with small interfering RNA (**Figures 2B, C**). After knocking down CDH2 expression, the proliferation ability of THCA cells TPC-1 and KTC-1 was significantly decreased (**Figures 2D, E**). Therefore, we conclude that CDH2 exerts its growth-promoting activity of THCA.

### CDH2-Related Genes in THCA

**Figure 3:**
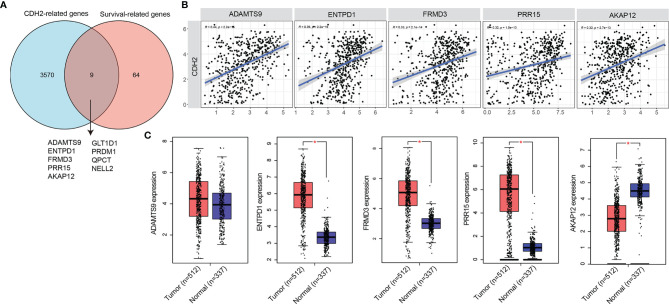
CDH2-related genes in THCA. **(A)** Venn diagram showed the intersecting part in CDH2-related genes and OS-related genes. **(B)** The correlation between CDH2 and ADAMTS9, ENTPD1, FRMD3, PRR15, AKAP12 showed a strong positive correlation. **(C)** The mRNA expression levels of ADAMTS9, ENTPD1, FRMD3, PRR15, AKAP12 of tumor tissues and normal tissues in THCA were generated by GEPIA database. *P < 0.05.

Next, we investigated how CDH2 was regulated in THCA. We first identified genes that were positively associated with CDH2 by pearson’s test (n=3575), and those correlating with OS in patients of THCA by COX test (n=73) (**Figure 3A**). The results of the Venn diagram showed that there are 9 genes in the intersecting part in **Figure 2A**, they were ADAMTS9, ENTPD1, FRMD3, PRR15, AKAP12, GLT1D1, PRDM1, QPCT, NELL2. The correlation between CDH2 and ADAMTS9, ENTPD1, FRMD3, PRR15, AKAP12 showed a strong positive correlation (R>0.3, P<0.0001) (**Figure 3B**). However, CDH2 exhibited a moderate association with GLT1D1, PRDM1, QPCT, NELL2 (R<0.3) (**Supplement Figure 1**).

We further verified the expression levels of ADAMTS9, ENTPD1, FRMD3, PRR15, AKAP12 in THCA, and the results of GEPIA database showed that ENTPD1, FRMD3, PRR15 were obviously increased in tumor tissues (n=512) compared with normal tissues (n=337), and the difference was statistically significant (**Figure 3C**). However, the mRNA levels of ADAMTS9 in tumor tissues were not statistically different from those in normal tissues (**Figure 3C**). While AKAP12 was significantly higher in normal tissues than in tumor tissues (**Figure 3C**).

In order to further confirm the correlation of ADAMTS9, ENTPD1, FRMD3, PRR15, AKAP12 with CDH2, we investigated their relevance to EMT-related genes (CDH1, CDH2, SNAI1, SNAI2, SNAI3, TWIST1, TWIST2, ZEB1, ZEB2, VIM). Interestingly, ADAMTS9, ENTPD1, FRMD3, PRR15, AKAP12 revealed strong correlations with EMT-related genes (**Supplement Figure 2**).

### The Binding MiRNAs of CDH2 and CDH2-Related Genes

**Figure 4:**
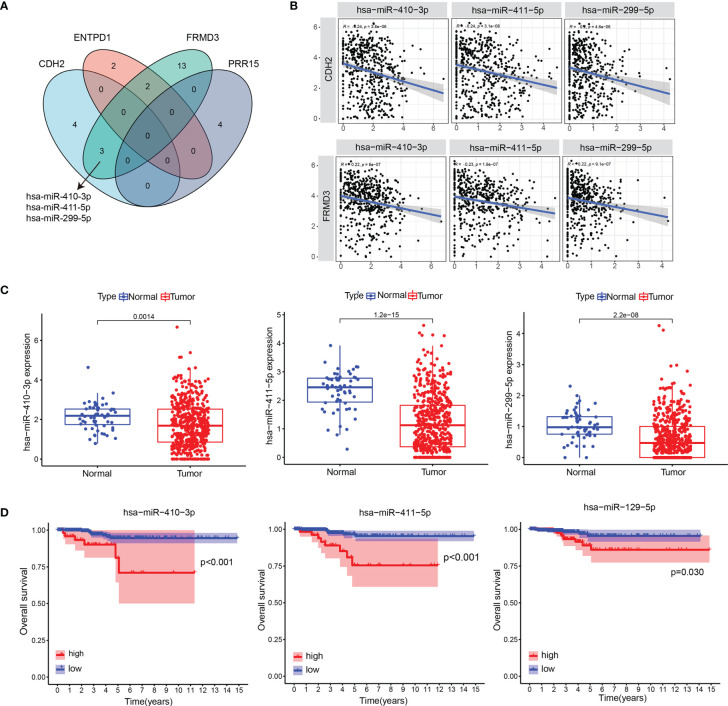
The binding miRNAs of CDH2 and CDH2-related genes. **(A)** Venn diagram showed the binding miRNAs of CDH2, ENTPD1, FRMD3 and PRR15. **(B)** The correlations between genes (CDH2, FRMD3) and miRNAs (hsa-miR-410-3p, hsa-miR-411-5p, hsa-miR-299-5p) were performed in TCGA dataset. **(C)** The levels of hsa-miR-410-3p, hsa-miR-411-5p and hsa-miR-299-5p in tumor tissues and normal tissues in THCA. **(D)** The association of hsa-miR-410-3p, hsa-miR-411-5p and hsa-miR-299-5p expression and OS of THCA patients in TCGA dataset.

We used the ENCORI database (https://starbase.sysu.edu.cn/) to survey the binding miRNAs of CDH2, ADAMTS9, ENTPD1, FRMD3, PRR15, AKAP12. Our data showed that only CDH2, ENTPD1, FRMD3 and PRR15 exhibited the required miRNAs, and the Venn diagram suggested that only FRMD3 had 3 intersecting miRNAs with CDH2, which were hsa-miR-410-3p, hsa-miR-411-5p, hsa-miR-299-5p (**Figure 4A**). Pearson correlation analysis revealed that there were negative correlations between genes (CDH2, FRMD3) and miRNAs (hsa-miR-410-3p, hsa-miR-411-5p, hsa-miR-299-5p) (**Figure 4B**). Furthermore, hsa-miR-410-3p, hsa-miR-411-5p and hsa-miR-299-5p were significantly decreased in tumor tissues compared with normal tissues in THCA (**Figure 4C**). In addition, hsa-miR-410-3p, hsa-miR-411-5p and hsa-miR-299-5p had predictive effects on the OS of THCA patients (**Figure 4D**).

### Identification of Genes and MiRNAs Expression in Clinical THCA Tissues

**Figure 5:**
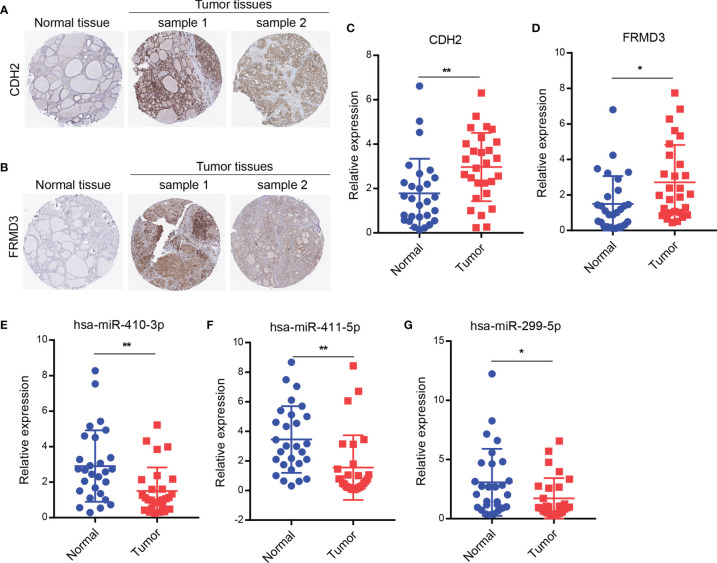
Identification of genes and miRNAs expression in clinical THCA tissues. **(A, B)** The protein expression of CDH2 or FRMD3 by IHC using the HPA database. **(C, D)** The mRNA levels of CDH2 or FRMD3 were checked in 28 paired clinically fresh THCA of carcinoma and paraneoplastic tissues by qRT-PCR assay. **(E–G)** The miRNA levels of has-miR-410-3p or has-miR-411-5p or has-miR-299-5p were measured in 28 paired clinically fresh THCA of carcinoma and paraneoplastic tissues by qRT-PCR assay. *P < 0.05; **P < 0.01.

To determine the expression levels of CDH2 and FRMD3, we next tested the protein expression of CDH2 and FRMD3 by IHC using the Human Protein Atlas database (HPA, https://www.proteinatlas.org/). Similar to the results of mRNA levels from the TCGA database, the protein expression levels of CDH2 and FRMD3 were significantly higher in tumor tissues of THCA than in normal tissues (**Figures 5A, B**). Next, we collected 28 paired clinically fresh THCA of carcinoma and paraneoplastic tissues, and the results showed that the mRNA expression of CDH2 and FRMD3 were significantly increased in tumor tissues compared with the paraneoplastic tissues by qRT-PCR assay (**Figures 5C, D**). Moreover, there were significant decreased of has-miR-410-3p, has-miR-411-5p and has-miR-299-5p in cancer tissues compared to normal tissues by qRT-PCR assay (**Figures 5E–G**). These results further confirmed that CDH2 and FRMD3 might be able to be potential biomarkers of THCA.

### The Functions and Pathways Associated With CDH2 and FRMD3

**Figure 6:**
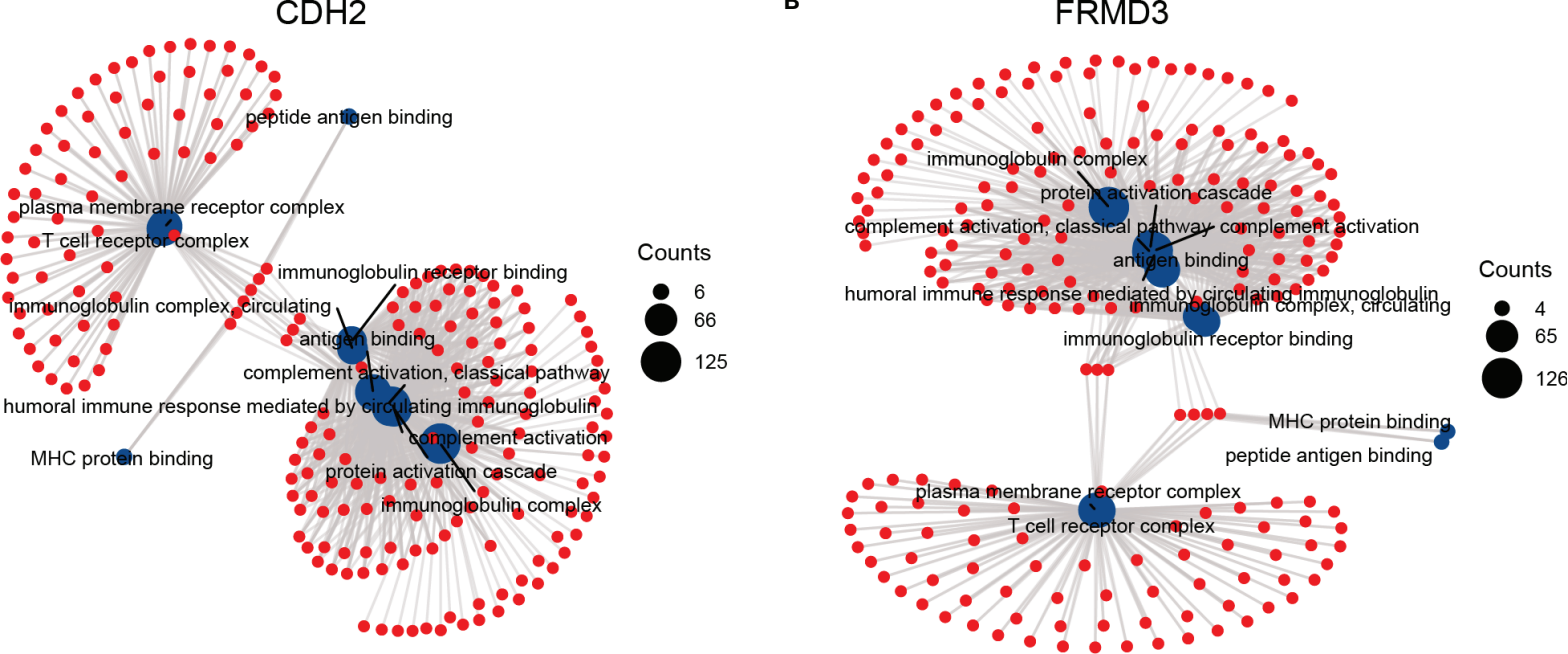
The functions and pathways associated with CDH2 and FRMD3. The Gene ontology (GO) and KEEG analysis were performed by the functions associated with CDH2 **(A)** and FRMD3 **(B)**.

Next, we investigated the related functions and signaling pathways of CDH2 and FRMD3 using Gene ontology (GO) and KEEG pathways. Interestingly, CDH2 and FRMD3 revealed very similar functional enrichment in BF (biological function), CC (cellular component), and MF (molecular function) (**Figures 6A, B**). In the BP, there were protein activation cascade, a humoral immune response mediated by circulating immunoglobulins and complement activation, the classical pathway (**Figures 6A, B**). In the CC, there were plasma membrane receptor complex, immunoglobulin complex, T cell receptor complex and immunoglobulin complex (**Figures 6A, B**). In the MF, there were MHC protein binding, peptide antigen binding, immunoglobulin receptor binding and antigen binding (**Figures 6A, B**). These results indicated that both CDH2 and FRMD3 showed to be very relevant to immunity.

### Correlation Between CDH2, FRMD3 and Immune Cell Infiltration in THCA

**Figure 7:**
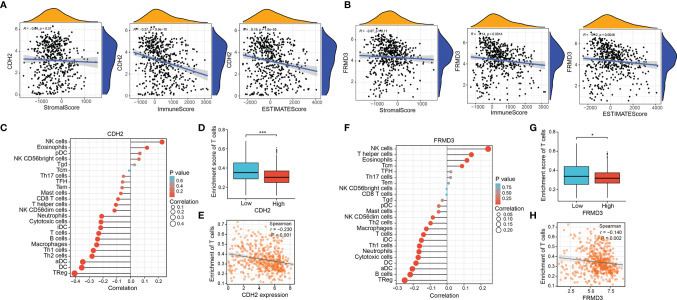
Correlation between CDH2, FRMD3 and immune cell infiltration in THCA. **(A, B)** The correlation of stromal scores, immune scores and ESTIMATE scores and CDH2 **(A)**, FRMD3 **(B)** expression and of TCGA dataset in THCA. **(C)** The relationship between CDH2 expression and immune cells was calculated by ssGSEA method in TCGA dataset. **(D)** The correlation between different CDH2 levels and T cell enrichment in TCGA dataset. **(E)** The correlation between CDH2 expression and T cell enrichment by spearman correlation analysis. **(F)** The relationship between FRMD3 expression and immune cells was calculated by ssGSEA method in TCGA dataset. **(G)** The correlation between different FRMD3 levels and T cell enrichment in TCGA dataset. **(H)** The correlation between FRMD3 expression and T cell enrichment by spearman correlation analysis. *P < 0.05; ***P < 0.001.

The above results showed that both CDH2 and were associated with immune function, therefore, we followed to explore whether CDH2 and FRMD3 played significant roles in the tumor microenvironment (TME). CDH2 expression levels were negatively correlated with immune score and ESTIMATE score; however, there was no association between CDH2 and stromal score (**Figure 7A**). Intriguingly, FRMD3 exhibited a similar behavior to CDH2 in the TME, that is, FRMD3 levels were negatively linked to immune scores and ESTIMATE scores, whereas FRMD3 was not related to stromal scores (**Figure 7B**). In addition, CDH2 and FRMD3 also demonstrated similar effects in immune cell infiltration. Specifically, high expression of CDH2 and FRMD3 resulted in the suppression of a large number of immune cells (**Figures 7C, F**). Moreover, there was a negative correlation between CDH2 and FRMD3 expression and T cell enrichment (**Figures 7D, G**). The T cell enrichment rate was significantly lower in the high CDH2 and FRMD3 expression groups than in the low expression groups (P < 0.05) (**Figures 7E, H**).

### Association of CDH2 and FRMD3 With Immune Checkpoints

**Figure 8:**
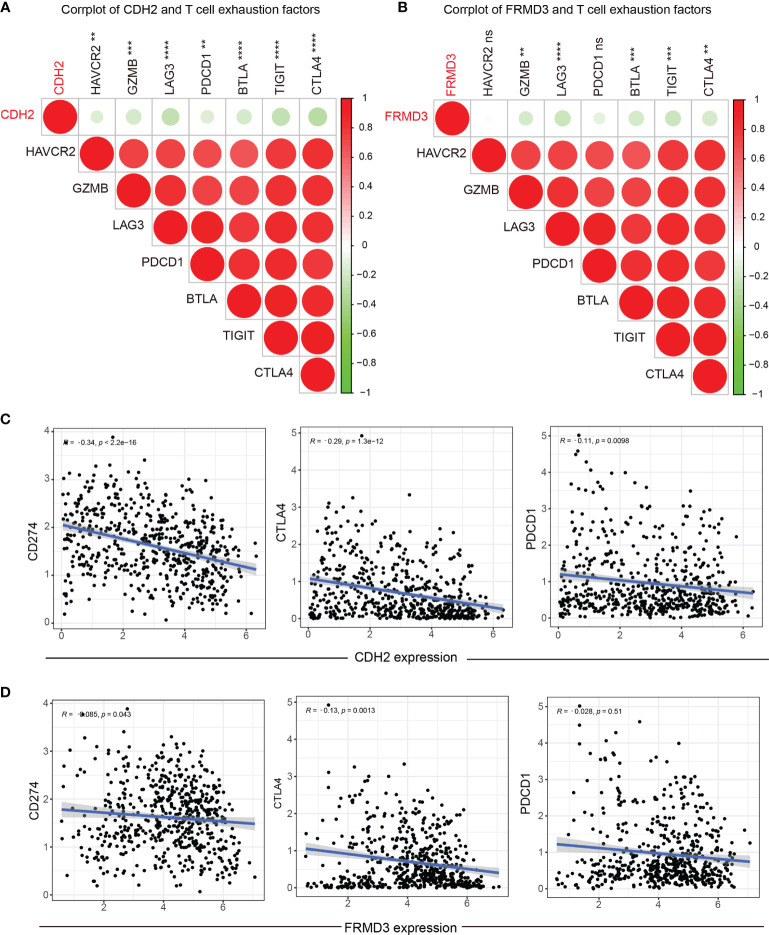
Association of CDH2 and FRMD3 with immune checkpoints. **(A, B)** Correlation between T cell exhaustion factors and CDH2 **(A)** and FRMD3 **(B)**. **(C, D)** The association between CD274, PDCD1, CTLA4 and CDH2 **(C)** and FRMD3 **(D)**. **P < 0.01; ***P < 0.001; ****P < 0.0001; ns, no significance.

As is known, T-cell exhaustion is critical for the effectiveness of immune checkpoint blockade and the achievement of adoptive T-cell transfer therapy (34). We further investigate the association between T-cell exhaustion marker genes and CDH2, FRMD3 levels. The results showed a strong negativity correlation between CDH2 and T-cell exhaustion factors (**Figure 8A**), and interestingly, FRMD3 showed a consistent role with CDH2 in T-cell exhaustion (**Figure 8B**). Moreover, CDH2 was negatively related to the immune checkpoint genes CD274, CTLA4 and PDCD1 (**Figure 8C**); similarly, FRMD3 was negatively correlated with CD274 and CTLA4, but not with PDCD1 (**Figure 8D**).

## Discussion

Currently, a minority of well-differentiated patients of THCA, as well as the majority of poorly differentiated thyroid carcinoma (PDTC) and anaplastic thyroid cancer (ATC) patients presenting with recurrence and metastasis, have become resistant to treatment with chemotherapy and radiotherapy (35). Thus, it has attracted extensive attention for researchers to motivate trials of new efficient therapies. A majority of cells with drug-resistant tend to exhibit an EMT phenotype in THCA. To understand the mechanical and cellular changes of this transient state as well as its relevance to the components of the microenvironment are hopeful to establish the underlying targets to overcome the recurrent and drug-resistant phenotype of tumors (36). Abnormal expression of the cell adhesion molecule N-cadherin is a hallmark of EMT, therefore, we explored the roles of CDH2 in THCA and its potential mechanisms. Here, we showed that CDH2 expression levels were significantly higher in tumor tissues than in normal tissues in THCA. Moreover, there were strongly associations of CDH2 mRNA expression with the stages T and N. We further explored the effects of CDH2 on the cell function of THCA, our data suggested that CDH2 exerted its growth-promoting activity of THCA. To better understand how CDH2 was regulated in THCA, we performed correlation analysis and verified that ADAMTS9, ENTPD1, FRMD3, PRR15, AKAP12 levels were associated with CDH2 and OS of THCA patients.

FRMD3 is presumed to be associated with susceptibility to diabetic nephropathy (37, 38), and in recent years, several reports have reported its role in tumors. The *FRMD3* gene was identified as a novel putative tumor suppressor in non-small cell lung cancer (33). However, high FRMD3 expression in patients with rectal carcinomatosis is associated with unfavorable prognosis (39). In this study, The mRNA and protein expression levels of FRMD3 were significantly elevated in THCA tissues, implying that FRMD3, similar to CDH2, plays a tumor-promoting function in THCA. To explore the regulatory mechanisms of CDH2 and FRMD3, we sought their common binding miRNAs. The pearson correlation analysis revealed that there were negative correlations between genes (CDH2, FRMD3) and miRNAs (hsa-miR-410-3p, hsa-miR-411-5p, hsa-miR-299-5p). Moreover, hsa-miR-410-3p, hsa-miR-411-5p and hsa-miR-299-5p were significantly decreased in tumor tissues compared with normal tissues in THCA. These results suggested that hsa-miR-410-3p, hsa-miR-411-5p, hsa-miR-299-5p might regulate both CDH2 and FRMD3, however, more cell function tests need to be validated.

GO and KEEG results showed that CDH2 and FRMD3 were strongly associated with immune-related functions, therefore, we considered that CDH2 and FRMD3 may play an important role in the tumor immune microenvironment. It is a hallmark of malignancies that cancer cells are able to escape immune disruption. Pro-inflammatory cytokines and chemokines are secreted by immune cells activating in the TME, thus promoting the growth of cancer cells (22). Cancers express specific antigens that are recognized by the major components of the immune response engaged in immune surveillance (40). It is considered that tumors can impair host immune cells within the TME and evade their surveillance by recruiting immune-suppressive cells, reducing tumor immunogenicity, or adopting other immune-suppressive mechanisms (22). THCA-associated inflammation presents a critical challenge for both diagnosis and new treatment strategies. Better understanding of the molecular and immunological features of TME is expected to enable the adoption of novel and more efficient immunotherapy approaches in advanced THCA. In this study, high expression of CDH2 and FRMD3 is highly linked to the suppression of immune cells.

ICP therapeutic monoclonal antibodies have changed the therapeutic prospective for many tumors, including THCA (23, 24). As a result, however, response rates are still relatively weak in the majority of cases. The lack or absence of tumor T-cell infiltration, a characteristic of so-called “cold tumors”, is a key factor associated with initial resistance to ICP inhibitors (41, 42). Therefore, it is vital to identify the mechanisms that lead to heat or cold immunity to tumors to enhance anti-tumor immunity. Our data indicated that CDH2 and CDH2-related gene FRMD3 might have the critical effects on altering tumors becoming ‘cold tumors’ eventually leading to immune checkpoint inhibitors resistance.

However, cellular and animal experiments should be performed to validate our results. In addition, further studies on large clinical samples are also needed.

## Conclusions

We revealed that the gene CDH2 may act as an essential function in tumorigenesis, immune cell infiltration of THCA. CDH2 and CDH2-associated gene FRMD3 may have key roles in altering tumors to become “cold tumors”, ultimately leading to resistance of immune checkpoints.

## Data Availability Statement

The datasets presented in this study can be found in online repositories. The names of the repository/repositories and accession number(s) can be found in the article/**Supplementary Material**.

## Ethics Statement

The studies involving human participants were reviewed and approved by the committee of Zhejiang Provincial People’s Hospital. The patients/participants provided their written informed consent to participate in this study. Written informed consent was obtained from the individual(s) for the publication of any potentially identifiable images or data included in this article.

## Author Contributions

YC designed the experiments, analyzed the results, prepared figures, authored, reviewed drafts of the manuscript, and approved the final draft. CH and QZ performed the experiments, prepared figures, and approved the final draft. ZQ designed the experiments, reviewed drafts of the manuscript, and approved the final draft. All authors contributed to the article and approved the submitted version.

## Funding

This project is supported by Zhejiang Provincial Medical and Health Science and Technology project (2017KY203); Zhejiang Provincial Natural Science Foundation of China (LQ18H160024).

## Conflict of Interest

The authors declare that the research was conducted in the absence of any commercial or financial relationships that could be construed as a potential conflict of interest.

## Publisher’s Note

All claims expressed in this article are solely those of the authors and do not necessarily represent those of their affiliated organizations, or those of the publisher, the editors and the reviewers. Any product that may be evaluated in this article, or claim that may be made by its manufacturer, is not guaranteed or endorsed by the publisher.

## Supplementary Material

Supplementary Figure 1 CDH2-related genes in THCA. The correlation between CDH2 and, GLT1D1, PRDM1, QPCT, NELL2 showed positive correlation.

Supplementary Figure 2 CDH2 and CDH2-related genes were correlated with EMT-related genes. Heatmap results showed that ADAMTS9, ENTPD1, FRMD3, PRR15, AKAP12 revealed strong correlations with EMT-related genes.

## Abbreviations

THCA, Thyroid cancer; EMT, Epithelial mesenchymal transition; TCGA, The Cancer Genome Atlas; ENCORI, Encyclopedia of RNA Interactomes; GEPIA, Gene Expression Profiling Interactive Analysis; GO, Gene Ontology; KEGG, Kyoto Encyclopedia of Genes and Genomes; DTC, Differentiated thyroid carcinoma; PTC, papillary thyroid carcinoma; FTC, follicular thyroid carcinoma; MTC, medullary thyroid carcinoma; FPKM, Fregments Per Kilobase per Million; TPM, transcripts per million reads; RPM, Reads per Million mapped reads; FPR, False Positive Rate; TPR, True Positive Rate; BRCA, Breast invasive carcinoma; OS, overall survival; BF, biological function; CC, cellular component; MF, molecular function; TME, tumor microenvironment; PDTC, poorly differentiated thyroid carcinoma; ATC, anaplastic thyroid cancer; RISC, RNA-induced silencing complex; ICP, Immune-checkpoint; DMEM, Dulbecco’s Modified Eagle Media (DMEM); FBS, Fetal Bovine Serum; CCK8, Cell Counting Kit-8.
