# Supplementary figures and images for: Roles of Cadherin2 in Thyroid Cancer
Source: Front Oncol. 2022 Jun 9;12:804287. doi: 10.3389/fonc.2022.804287 (PMC9218104; doi:10.3389/fonc.2022.804287)

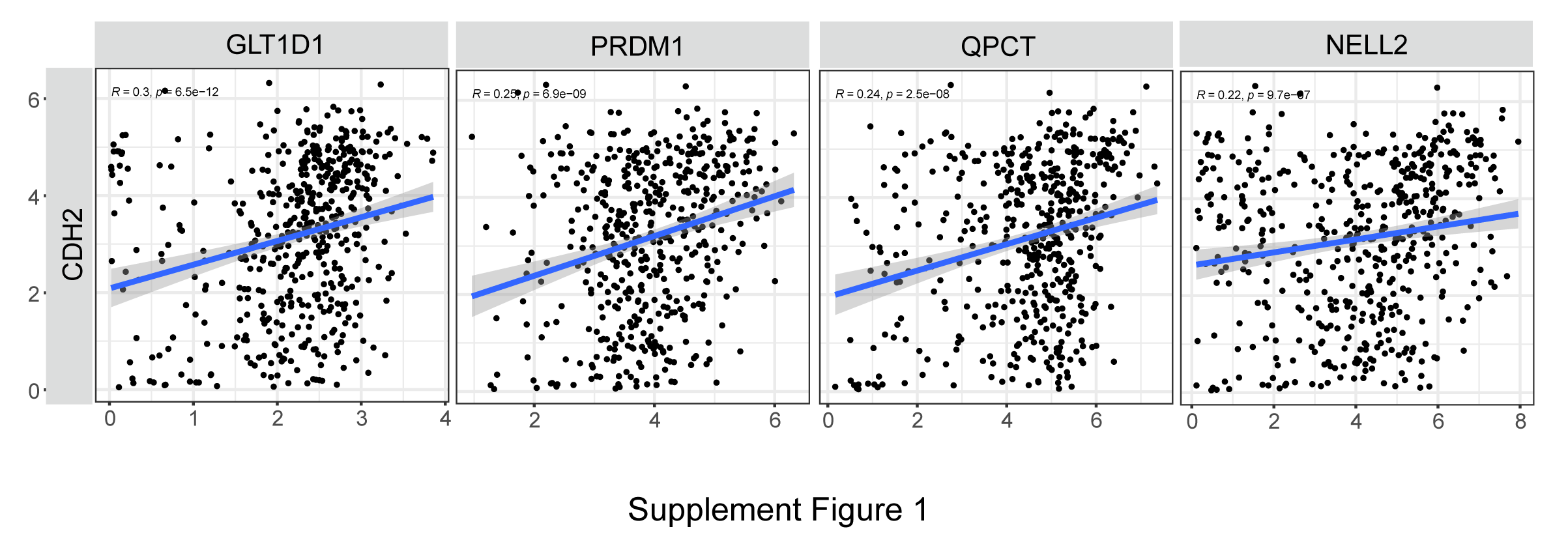

Supplement: Supplementary Figure 1 — CDH2-related genes in THCA. The correlation between CDH2 and, GLT1D1, PRDM1, QPCT, NELL2 showed positive correlation. [file Image_1.tif]

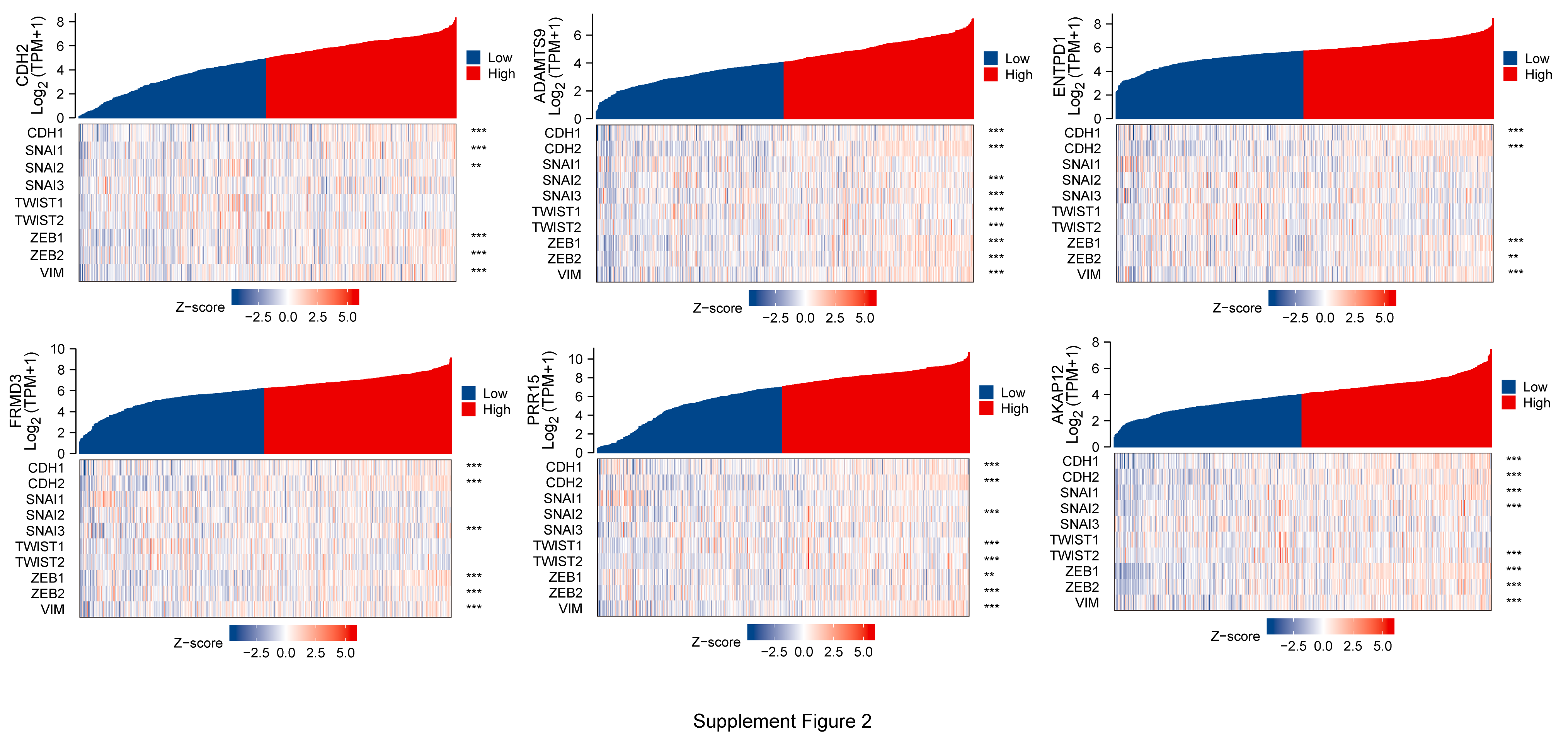

Supplement: Supplementary Figure 2 — CDH2 and CDH2-related genes were correlated with EMT-related genes. Heatmap results showed that ADAMTS9, ENTPD1, FRMD3, PRR15, AKAP12 revealed strong correlations with EMT-related genes. [file Image_2.tif]
