# Supplementary material for: Roles of Cadherin2 in Thyroid Cancer
Source: Front Oncol. 2022 Jun 9;12:804287. doi: 10.3389/fonc.2022.804287 (PMC9218104; doi:10.3389/fonc.2022.804287)
Supplement: Supplementary file 3 [file Table_1.docx]

| **Parameters** | **Total (N)** |
| --- | --- |
| Age (years) |  |
| <50 | 8 |
| ≥50 | 20 |
| Gender |  |
| Female | 11 |
| Male | 17 |
| Histological subtype |  |
| Papillary thyroid carcinoma (PTC) | 17 |
| Follicular thyroid carcinoma (FTC) | 8 |
| Medullary thyroid carcinoma (MTC) | 2 |
| Undifferentiated thyroid carcinoma | 1 |
| TNM stage |  |
| I | 21 |
| II | 6 |
| III-IV | 1 |
| Nodal Metastasis status |  |
| N0 | 19 |
| N1 | 9 |
| M stage |  |
| M0 | 27 |
| M1 | 1 |

Supplement Table 1: The clinicopathological features of THCA patients.
